# 

**Published:** 1938

**Authors:** 


Vol. LV. No. 209 AUTUMN, 1938.

INDEXED

### The Bristol Medico-Chirurgical Journal

PUBLISHED UNDER THE AUSPICES OF

THE BRISTOL MEDICO-CHIRURGICAL SOCIETY.

*Editor:* J. A. NIXON, C.M.G., M.D., F.R.C.P.

WITH WHOM ARE ASSOCIATED

E. W. HEY GROVES, D.Sc., M.S., F.R.C.S.

A. E. ILES, O.B.E., M.B., B.S., F.R.C.S.

A. RENDLE SHORT, M.D., B.S., B.Sc., F.R.C.S.

E. WATSON-WILLIAMS, M.C., Ch.M., F.R.C.S., *Assistant Editor.*

*Editorial Secretary:* A. L. FLEMMING, M.B., Ch.B., D.A.

BRISTOL: J. W. ARROWSMITH LTD., 11 QUAY STREET

London: J. W. Arrowsmith (London) Ltd.
Fulwood House, Fulwood Place, High Holborn, W.C. 1

**Price Three Shillings net.**

PRINTED IN GREAT BRITAIN.

